# Fecal and vaginal microbiota of vaccinated and non-vaccinated pregnant elk challenged with *Brucella abortus*

**DOI:** 10.3389/fvets.2024.1334858

**Published:** 2024-01-30

**Authors:** Bienvenido W. Tibbs-Cortes, Faith M. Rahic-Seggerman, Stephan Schmitz-Esser, Paola M. Boggiatto, Steven Olsen, Ellie J. Putz

**Affiliations:** ^1^Infectious Bacterial Diseases Research Unit, United States Department of Agriculture, Ames, IA, United States; ^2^Interdepartmental Microbiology Graduate Program, Iowa State University, Ames, IA, United States; ^3^Department of Animal Science, Iowa State University, Ames, IA, United States

**Keywords:** brucellosis, microbiome, elk, RB51, pregnancy, fecal, vaginal

## Abstract

**Introduction:**

*Brucella abortus* is the causative agent of brucellosis in cattle and in humans, resulting in economic losses in the agricultural sector and representing a major threat to public health. Elk populations in the American Northwest are reservoirs for this bacterium and transmit the agent to domestic cattle herds. One potential strategy to mitigate the transmission of brucellosis by elk is vaccination of elk populations against *B. abortus*; however, elk appear to be immunologically distinct from cattle in their responses to current vaccination strategies. The differences in host response to *B. abortus* between cattle and elk could be attributed to differences between the cattle and elk innate and adaptive immune responses. Because species-specific interactions between the host microbiome and the immune system are also known to affect immunity, we sought to investigate interactions between the elk microbiome and *B. abortus* infection and vaccination.

**Methods:**

We analyzed the fecal and vaginal microbial communities of *B. abortus*-vaccinated and unvaccinated elk which were challenged with *B. abortus* during the periparturient period.

**Results:**

We observed that the elk fecal and vaginal microbiota are similar to those of other ruminants, and these microbial communities were affected both by time of sampling and by vaccination status. Notably, we observed that taxa representing ruminant reproductive tract pathogens tended to increase in abundance in the elk vaginal microbiome following parturition. Furthermore, many of these taxa differed significantly in abundance depending on vaccination status, indicating that vaccination against *B. abortus* affects the elk vaginal microbiota with potential implications for animal reproductive health.

**Discussion:**

This study is the first to analyze the vaginal microbiota of any species of the genus *Cervus* and is also the first to assess the effects of *B. abortus* vaccination and challenge on the vaginal microbiome.

## Introduction

The Rocky Mountain elk (*Cervus canadensis nelsoni*) is a large ruminant of the family Cervidae found across North America. In 2017 more than 125,000 elk were estimated to populate the Greater Yellowstone Area (GYA), defined as Yellowstone and Grand Teton National Parks and surrounding areas in Idaho, Wyoming, and Montana (1). In the United States, elk in the GYA represent one of the two remaining reservoirs in the United States of the bacterium *Brucella abortus*, the causative agent of bovine brucellosis. As such these elk pose a risk to domestic cattle and other livestock (1). Supplemental feeding of elk occurs during winter months in the GYA as part of conservation efforts, but this has led to increased disease transmission within supplementally fed elk herds (2, 3). In addition to the risk of brucellosis transmission posed by these winter-fed populations to domestic cattle, research also indicates that free-roaming, unfed elk populations have interacted with cattle and caused bovine brucellosis outbreaks (2–5).

Brucellosis in cattle is primarily characterized by late-term abortions due to the marked tropism of *B. abortus* for fetal and placental tissues. Transmission readily occurs via the mucosal route when an animal is exposed to fluids and tissues associated with the birth of an infected fetus as these materials can contain high bacterial loads of up to 10^10^ colony-forming units per gram (6). Brucellosis can also result in infertility due to metritis and orchitis, further contributing to animal health and economic burden (6–9). In addition to its effects on domestic livestock production, *B. abortus* is zoonotic and can cause chronic and debilitating disease in humans. Human infection is characterized by chronic bouts of fever, malaise, inflammation, and osteoarticular complications (10, 11). Due to the economic costs of *B. abortus* infection in cattle and the zoonotic threat to humans, millions of dollars have been invested in eradicating brucellosis in the United States.

Evidence indicates that elk are the predominant source of transmission for *B. abortus* to cattle in the GYA, and multiple occurrences of elk-to-cattle spread have been documented (1, 4, 5, 12–14). One solution to help mitigate the spread of brucellosis to domestic cattle is brucellosis vaccination of cattle and free-ranging elk. While the rough *B. abortus* strain RB51 (RB51) vaccine is efficacious in cattle (15–18), research indicates that it induces poor cellular immune responses in elk and lacks efficacy in preventing infection or abortion after experimental challenge (19, 20). In addition to the differential response to RB51, elk also respond differently from cattle to *B. abortus* challenge. Data from standardized experimental challenge studies suggest that *B. abortus* infection causes a lower abortion rate in elk when compared to cattle, with some variance reported between studies (15, 21–26). Host-specific differences in host-pathogen interactions may result from differences in innate or adaptive immunity among other factors. Due to the interactions between the microbiome and the host immune system, variations in microflora between species may drive differences in host interactions with *B. abortus* and other pathogens (27–30).

While brucellosis is widely associated with abortion and reproductive problems in large animals, previous work has reported that brucellosis infection induces changes in the gut microbiota of mice (31, 32). Therefore, we hypothesized that *B. abortus* infection might induce changes in the fecal and vaginal microflora of elk; we also hypothesized that these microbial communities might be affected by vaccination of elk with RB51. To our knowledge, no studies have investigated the effects of *B. abortus* infection on the host vaginal microbiota. Additionally, although the gut and vaginal microbiomes of ruminants including cattle and sheep have been extensively studied, there has been relatively limited analysis of *Cervus* (including *C. canadensis nelsoni*) gut microbiota. Furthermore, no characterization of the vaginal microbiota of any *Cervus* species has been conducted. We were therefore interested in understanding how virulent *B. abortus* challenge and RB51 vaccination altered the fecal and vaginal microbiota of periparturient elk. These data expand knowledge of the effects of *B. abortus* infection on the host mucosal environment and provide insight into the composition and community structure of the elk vaginal microbiota.

## Materials and methods

### Elk care and sampling

**Figure 1:**
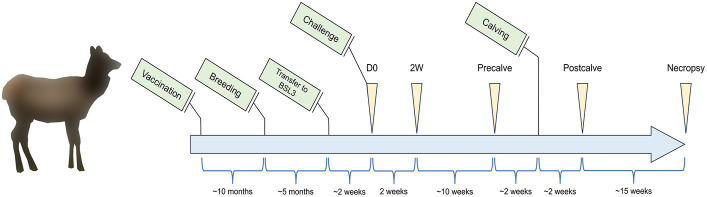
Study timeline listing events (green boxes), sampling timepoints (yellow triangles), and the time between events and sampling timepoints (blue brackets). Elk cows (*n* = 16) between the ages of 8–10 months were enrolled in the study and vaccinated with *Brucella abortus* RB51 (*n* = 8) or unvaccinated as a control group (*n* = 8). Elk were subsequently bred 10 months post-vaccination before being moved to a BSL3 facility at the beginning of the third trimester. After an acclimation period, elk cows were challenged with *B. abortus* 2308. The fecal and vaginal microbiota of the elk cows were subsequently sampled at the indicated timepoints: D0—time of challenge, 2W—2 weeks after challenge, Precalve—the closest timepoint to 2 weeks prior to calving, Postcalve—approximately 2 weeks following natural calving, and Necropsy—14–17 weeks postpartum at necropsy. Tissue samples for *B. abortus* cultures were conducted at Necropsy. Figure is not to scale.

This study was carried out in accordance with protocols approved by the National Animal Disease Center (NADC) Institutional Animal Care and Use Committee. A diagram of the study timeline is provided in Figure 1 showing major events relevant to the study (green boxes), sampling times (yellow arrows), and the time period between events and sample collections (blue brackets). Sixteen elk hinds were purchased from a brucellosis-free herd and housed in outdoor field barns at the NADC campus in Ames, IA. Elk were fed approximately 3 pounds of commercially available Trophy Image 20 pellet feed (Kent Nutrition Group) per head daily while on pasture with access to free choice long stem grass hay. At approximately 1.5–2 years of age, animals were randomly assigned to either the cohort of non-vaccinate controls (*n* = 8) or the cohort of RB51-vaccinated animals (*n* = 8). Control animals received 2 mL of sterile saline delivered subcutaneously. RB51 vaccinates received 2 × 10^10^ colony forming units of a commercial RB51 vaccine (Colorado Serum Company), administered subcutaneously. Approximately 10 months after vaccination, elk were pasture bred by natural cover. Pregnancy status was assessed via a commercial enzyme linked immunosorbent assay (ELISA) for the serological detection of pregnancy-specific protein B (Biotracking Inc.). At approximately mid-gestation, elk were moved into a BSL3 high containment facility and were given a 2–3 week acclimation period before challenge. Elk continued to receive the same ration of pellet feed with the only change in diet being the replacement of pasture and free choice hay with free choice Alfalfa-Timothy Cubes (Ontario Dehy). Animals had constant access to city treated water, and the diet described above was kept constant for the duration of the study. For challenge, elk were anesthetized intramuscularly with xylazine (0.5–0.7 mg/kg) and ketamine (2.0–2.5 mg/kg). Approximately 2.5 × 10^7^ colony forming units of *B. abortus* strain 2308 were administered to each animal via bilateral conjunctival inoculation, and anesthesia was reversed with intramuscular administration of tolazoline (1.0–1.1 mg/kg). Most sample equipment was single use and sterilely prepared, and all other instruments were autoclaved and sterilely maintained moving through containment.

The above anesthesia protocol was also used for sample collection. Samples were collected in accordance with Figure 1. Vaginal samples were collected by inserting sterile single use swabs approximately 10 cm into the vaginal canal by trained personnel taking care to avoid outer surface contamination. Vaginal swabs were stored in sterile 1× phosphate-buffered saline (PBS) and frozen at −80°C until subsequent DNA isolation. Fecal samples were collected when possible from the rectum of individual animals and frozen at −80°C in 50 mL conical tubes until further analysis.

All animals remained free of clinical signs throughout the study. All elk successfully calved naturally unassisted with the exception of one vaccinated animal with dystocia that required euthanasia. Because the animal underwent cervical dilation, rupture of membranes, and partial delivery of the calf through the birth canal, we retained its Postcalve timepoint in the dataset. Five of the elk cows were euthanized 1–3 weeks after parturition with variable fecal and vaginal collection. The remaining 11 cows (six unvaccinated and five vaccinated) were subsequently euthanized and necropsied approximately 31 weeks after challenge (14–17 weeks after parturition). Culture-based detection of *B. abortus* 2308 was performed on samples of elk lung, liver, spleen, and urogenital tract tissues as previously described (33). Briefly, tissues were homogenized in a 0.85% NaCl solution and incubated on tryptic soy agar with 5% bovine serum at 37°C, 5% CO_2_ for 72 h. Recovered colonies were confirmed as *B. abortus* 2308 using previously described PCR methods (33).

Successful challenge was confirmed with *B. abortus* 2308 culture detection in all animals except one RB51-vaccinated animal necropsied 1–3 weeks postpartum and one unvaccinated animal necropsied 14–17 weeks postpartum. A total of 154 samples were successfully sequenced of which 18 were negative controls. Of the 136 biological samples, 71 were vaginal samples and 65 were fecal samples. The distribution of these samples among treatment groups and collection times is shown in Table 1.

**Table 1:** Distribution of sequenced samples used in data analysis.

Sample type	Vaccination status	D0	2W	Precalve	Postcalve	Necropsy
Vaginal	Control	8	8	8	6	6
	RB51-vaccinated	8	8	8	8	3
Fecal	Control	7	7	7	6	6
	RB51-vaccinated	6	8	7	6	5

D0, Samples collected at the time of challenge; 2W, Samples collected 2 weeks after challenge; Precalve, Samples taken at the closest timepoint to 2 weeks prior to calving; Postcalve, Samples collected most immediately following natural calving; and Necropsy, Samples collected from necropsied animals 14–17 weeks postpartum.

### DNA extraction and 16S rRNA gene amplicon sequencing

The final samples selected for subsequent DNA extraction and analysis were divided into groups as follows: D0—samples collected immediately prior to challenge, 2W–samples collected 2 weeks after challenge, Precalve—samples taken at the closest timepoint to 2 weeks prior to calving, Postcalve—samples collected most immediately following natural calving, and Necropsy—samples collected from necropsied animals 14–17 weeks post-calving (Figure 1).

Vaginal swabs stored in sterile 1× PBS were thawed and vortexed to release biological materials from the swabs into the liquid. Subsequently, tubes were centrifuged at 4,696 × *g* for 3 min to pellet vaginal material. Frozen fecal samples were thawed, and 0.2 g of each sample were weighed out into a microcentrifuge tube. An additional 10 DNA extraction negative controls were conducted following the RIDE standards to identify contaminants in the experiment (34). Control samples consisted of PBS from unused vaginal swab tubes (four samples) and water added to unused fecal collection tubes (six samples).

DNA was extracted from the vaginal swab pellets, fecal samples, and DNA extraction controls using the DNeasy PowerLyzer Powersoil Kit (Qiagen) following the manufacturer’s instructions and including the optional 4°C incubation steps. Mechanical lysis was performed with a Vortex Genie 2 Model G-560 (Daigger) equipped with a Vortex Adapter (Qiagen) at maximum speed for 10 min. Extractions were conducted in a BSL3 lab following all relevant protocols for working with samples potentially contaminated with *B. abortus*. The concentration of the extracted DNA was measured using the Qubit dsDNA BR Assay Kit (Thermo Fisher), and DNA samples were stored at −20°C until submission for library preparation and sequencing.

DNA samples were submitted to the Iowa State University Genomics Core facility on two sterile 96-well plates for library preparation and sequencing. In addition to the 10 DNA extraction negative controls, eight wells (four per plate) were filled with diethyl pyrocarbonate-treated water. These negative controls were used to account for contamination introduced in the library preparation and sequencing process (34). Samples underwent custom library preparation for amplification of the 16S rRNA gene V4 region following a modified version of the 16S Illumina amplicon protocol created by the Earth Microbiome Project.^1^ The following primers were used: 515F—5′ GTGYCAGCMGCCGCGGTAA 3′ and 806RB: 5′ GGACTACNVGGGTWTCTAAT 3′. Modifications to the protocol consisted of the following: (1) use of a single amplification step for each sample instead of a triplicate amplification, (2) quantification of PCR product by a Quant-iT PicoGreen dsDNA Assay Kit (Invitrogen), (3) pooling equal amounts of amplicon from each sample (240 ng) and subsequent purification with a UltraClean PCR Clean-Up Kit (MO BIO Laboratories), (4) and utilization of a Mantis liquid handler (Formulamatrix) for processing. Paired-end 500-cycle sequencing was then performed on processed libraries using the Illumina MiSEQ platform.

### Generation and processing of OTUs from raw reads

Raw reads were analyzed with FASTQC for general quality, and a single sample representing feces collected from an RB51-vaccinated animal at necropsy was removed from the analysis due to poor sequencing quality. Using mothur (v1.43.0), the raw paired-end reads were merged and filtered to generate contigs. Contigs which were shorter than 252 bases, contained any ambiguities, and possessed homopolymeric regions greater than 8 bases were removed from the dataset. The remaining reads were then aligned against the SILVA SSU NR database alignment (v138) provided on the mothur website^2^ (35). More than 97.5% of the reads aligned within the same region, and reads which fell outside the bounds of this alignment were discarded. Chimeric sequences were subsequently removed using the SILVA.gold database provided on the mothur website as a reference (35). Finally, *de novo* operational taxonomic unit (OTU) clustering was performed on the remaining reads with a similarity threshold of 99%, and OTUs were classified using the aforementioned SILVA SSU NR database (v138). Output from mothur was next imported into R (v4.3.1) and processed using the package phyloseq (36). The package decontam was next used to identify OTUs which were likely the result of contamination and not genuine OTUs from the elk microbiota. A *P* score representing the likelihood that an OTU was a contaminant was first calculated for all OTUs using the prevalence method. *P* scores were then visualized and used to choose a classification threshold of 0.575 below which OTUs were classified as contaminants; contaminant OTUs were then removed from the dataset (37). Subsequently, OTUs represented by <10 reads were removed in order to reduce the prevalence of spurious OTUs resulting from PCR and sequencing errors.

### Alpha and beta diversity and differentially abundant taxa

Alpha diversity, beta diversity, relative abundance, and differential abundance were all calculated separately for fecal and vaginal samples. Alpha diversity was estimated accounting for both timepoint and vaccination status via divnet-rs,^3^ a Rust implementation of the R package DivNet which uses a log-ratio model to improve estimates (38). Shannon and Gini-Simpson indices were then calculated from the alpha diversity estimates. For both indices, the “betta_lincom” function was used to calculate differences in index between samples of a given vaccination status and timepoint, hereafter referred to as vaccination-timepoint (VT) combinations (39, 40). Multiple testing correction was performed with the Benjamini-Hochberg method, and indices were considered significantly different when the corrected *p* value was <0.05. Finally, the Shannon and Gini-Simpson indices were converted to Hill numbers (41). To visualize beta diversity, principal coordinates analysis (PCoA) was conducted in phyloseq using Bray–Curtis dissimilarity. For general assessment of abundance (but not identification of differentially abundant taxa), relative abundance was calculated for all phyla, families, genera, and OTUs using the package corncob (42). From this data a list of OTUs with >0.01% relative abundance was generated for the unvaccinated fecal, vaccinated fecal, unvaccinated vaginal, and vaccinated vaginal communities. These were then used to create a Venn diagram displaying the number of OTUs with >0.01% in the four communities.

The following model was used to evaluate the effects of time and vaccination status on the abundance (as calculated from raw read counts) of individual taxa:


$$Y_{ijk}=\mu +\upsilon_{i}+\tau_{j}+\upsilon_{i}\;\tau_{j}+\varepsilon_{ijk}$$

Where Y*_ijk_* is the observed value for *k*th experimental unit within the *i*th level of vaccination status (vaccinated vs. unvaccinated) at the *j*th timepoint (D0, 2W, Precalve, Postcalve, or Necropsy); μ is the overall mean; υ*_i_* is the fixed effect of the *i*th vaccination status (*i* = vaccinated vs. unvaccinated); τ*_j_* is the fixed effect of the *j*th timepoint (*j* = D0, 2W, Precalve, Postcalve, or Necropsy); τ*_i_* υ*_j_* is the interaction of vaccination status and timepoint; and *ε_ijk_* is the error as described by the model for *Y*_ijk_ (*k* = 1–16). Animal ID was included as a repeated measures random effect to account for the covariance among the samples taken from the same animal. Changes in the abundance of individual phyla, genera, and OTUs were analyzed using a negative binomial distribution in the GLIMMIX procedure in SAS (Version 9.4, SAS Inst., Cary, NC, United States) following the model described above (43). All phyla (18 in the fecal dataset and 15 in the vaginal dataset) were analyzed using the above methods, but only the 300 most abundant OTUs and the 100 most abundant genera for each sample type were evaluated. Fecal and vaginal samples were analyzed separately. The MULTTEST procedure within SAS was implemented to control for false discovery rate, and taxa were considered differentially abundant if they were classified as significant (*q* < 0.05) (44). For differentially abundant taxa, the log_2_fold changes between treatment groups were calculated and plotted using R.

### BLAST methods for additional taxonomic classification of OTUs

In some instances, mothur was unable to classify an OTU to the genus level. Therefore, all OTUs (including contaminants) represented by ≥10 reads were further analyzed using BLAST+ (v2.13.0) (45). The “get.oturep” command in mothur was used to retrieve a representative sequence for each OTU, and the entire 16S rRNA RefSeq nucleotide database (containing 27,020 16S rRNA gene sequences) was downloaded on August 16, 2023 (46). The 16S rRNA RefSeq database was converted into a BLAST database using “makeblastdb.” BLAST was then run with the OTU representative sequences as query against the 16S BLAST database using the “blastn” algorithm with “word_size” set to 6 and “max_hsps” set to 2. The top 10 matches in terms of *e*-value were kept for each query. Taxa in this manuscript are referred to by their mothur classification unless explicitly stated.

### Identification of *Brucella abortus* reads from a *Brucella abortus* microbiome sequencing experiment in mice

Han et al. (32) performed a microbiome analysis on feces of mice challenged with *B. abortus* 2308 via the oral route. The authors successfully cultured *B. abortus* from the gut tissues of the infected mice but did not explicitly state whether they were able to identify reads classified as *B. abortus* in their 16S rRNA gene amplicon data. To provide context for our study, we sought to determine if *B. abortus* DNA was detected in the aforementioned experiment by using the *B. abortus* strain 2308 (AM040265.1) 16S rRNA gene sequence as a BLAST query against the SRA data of Han et al (SRP427299). Using the Sequence Read Archive Nucleotide BLAST tool,^4^ the 30 SRA experiment sets were set as the reference, and blastn was run with default parameters. A 98% percent identity threshold was adopted to identify amplicon reads derived from the *B. abortus* 16S rRNA gene.

## Results

### Results of 16S rRNA gene amplicon sequencing and mothur

A total of 10,901,191 raw reads were generated by Illumina MiSeq. Quality control, alignment, and chimera removal in mothur reduced this number to 6,718,641 high-quality reads, and *de novo* OTU generation at 99% sequence similarity yielded 229,959 OTUs. 1,292 of these OTUs were classified as contaminants by Decontam. These were removed from the dataset and can be found in Supplementary File 1. 216,350 OTUs were represented by <10 reads in biological samples and were discarded, resulting in a final dataset of 12,317 OTUs. All OTUs represented by ≥10 reads as well as their mothur classifications and decontam *P* scores are listed in Supplementary File 1.

### The community structure of the elk vaginal microbiota is more variable than that of the fecal microbiota

**Figure 2:**
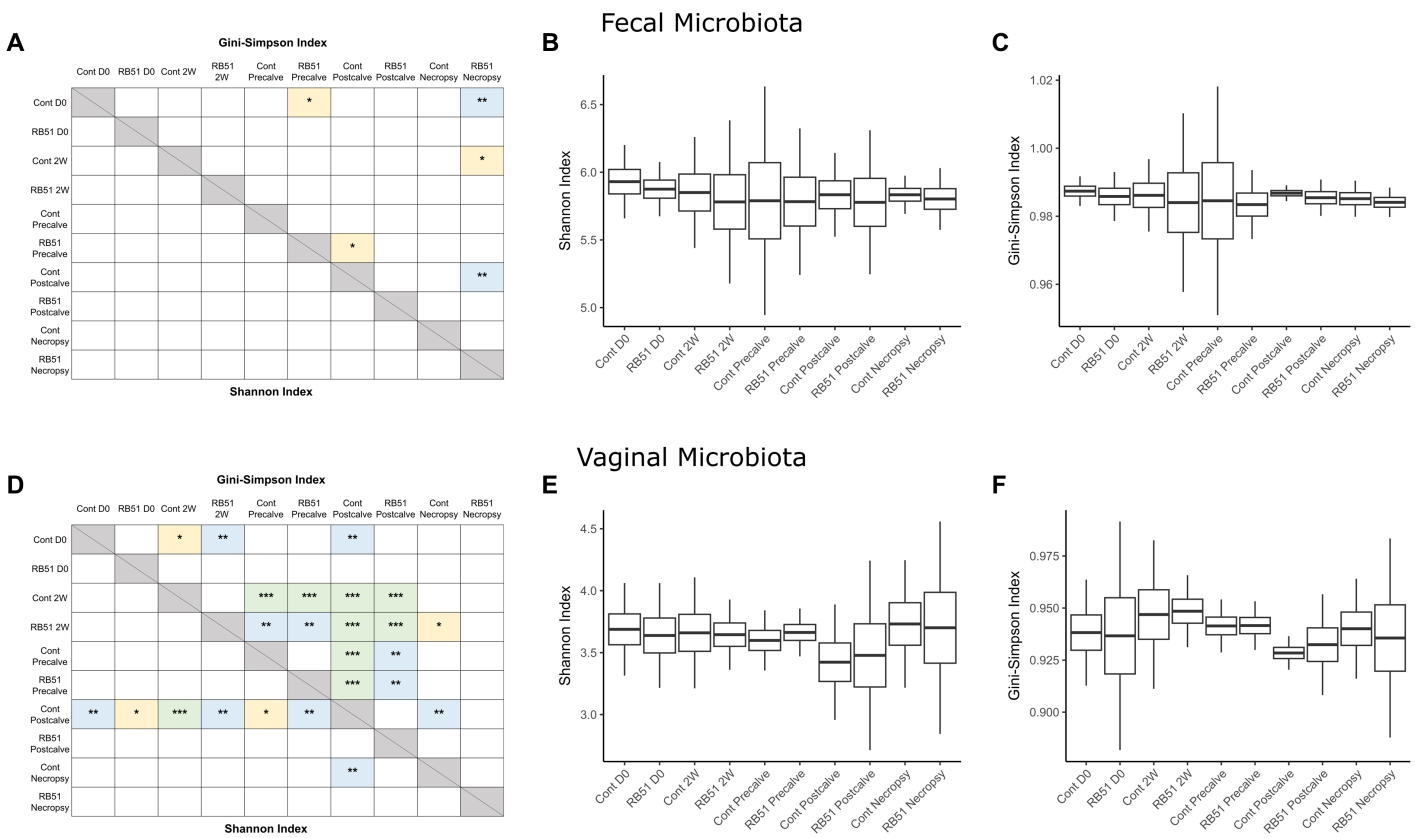
Comparisons of alpha diversity between elk cow samples of a given VT combination; panels **(A–C)** represent fecal samples while panels **(D–F)** represent vaginal samples. Panels **(A,D)** display the significance of pairwise comparisons between the alpha diversity indices of each VT combination (^*^p < 0.05, ^**^p < 0.01, ^***^p < 0.001). Shannon index comparisons are shown in the lower triangle while Gini-Simpson index comparisons are shown in the upper triangle. Panels **(B,C,E,F)** display box plots showing the mean alpha diversity index for each VT combination. Cont, Samples from unvaccinated controls; RB51, Samples from RB51-vaccinated animals.

**Figure 3:**
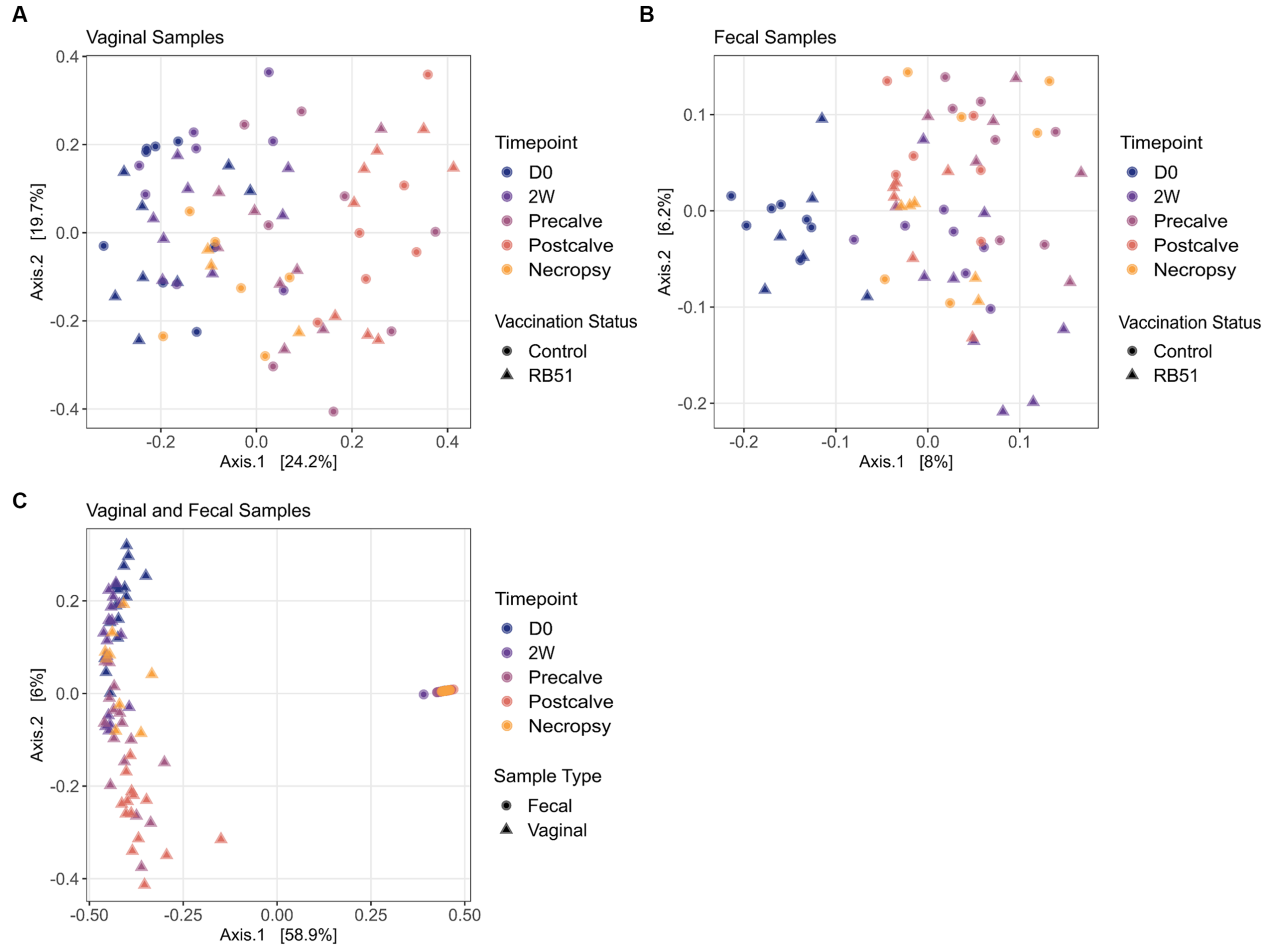
Principal Coordinates Analysis (PCoA) plots for each vaginal sample (panel **A**) and fecal sample **B**). Panel **(C)** shows a PCoA plot of all samples in the experiment. PCoA plots are based on Bray–Curtis dissimilarity. Samples are colored by timepoint and are differentiated in vaccination status by shape. RB51, Samples from RB51-vaccinated animals.

Alpha diversity indices and corresponding Hill numbers are listed for each VT combination in Table 2. Figures 2A,D display the results of comparisons of alpha diversity index between each VT combination; Gini-Simpson index comparisons are displayed in the upper right triangle while the comparisons of Shannon index are displayed in the lower left triangle. Box plots of the alpha diversity indices for each VT combination are shown in Figures 2B,C,E,F. For fecal samples, the Shannon indices of all VT combinations were not significantly different from each other. However, some fecal VT combinations had significantly different alpha diversities as measured by the Gini-Simpson index (Figure 2A). These included the RB51-necropsy communities which were less diverse compared to the communities of control animals at D0, 2W, and Postcalve. PCoA analysis showed that the fecal samples of different vaccination status and timepoint were not clearly separated by the first two principal coordinates (Figure 3B). Fecal and vaginal communities were also clearly separated from each other (Figure 3C). This stark difference was further demonstrated by the fact that only four OTUs had a relative abundance >0.01% in both the fecal and vaginal communities (Supplementary Figure 1).

**Table 2:** Alpha diversity indices and Hill numbers (effective species) for each treatment.

	Fecal microbiota				Vaginal microbiota			
Treatment	Shannon	Hill-Shannon	Gini-Simpson	Hill-Simpson	Shannon	Hill-Shannon	Gini-Simpson	Hill-Simpson
Control D0	5.930	376.3	0.987	79.3	3.688	40.0	0.938	16.2
Vaccinated D0	5.875	356.1	0.986	70.6	3.638	38.0	0.937	15.8
Control 2W	5.850	347.3	0.986	72.2	3.660	38.9	0.947	18.8
Vaccinated 2W	5.781	324.0	0.984	62.6	3.645	38.3	0.948	19.4
Control precalve	5.789	326.8	0.985	64.7	3.599	36.5	0.941	17.1
Vaccinated precalve	5.783	324.7	0.983	60.4	3.663	39.0	0.942	17.1
Control postcalve	5.834	341.6	0.987	75.6	3.423	30.7	0.928	14.0
Vaccinated postcalve	5.778	323.0	0.985	68.7	3.478	32.4	0.932	14.8
Control necropsy	5.834	341.6	0.985	67.4	3.731	41.7	0.940	16.7
Vaccinated necropsy	5.803	331.2	0.984	62.9	3.701	40.5	0.936	15.5

D0, Samples collected at the time of challenge; 2W, Samples collected 2 weeks after challenge; Precalve, Samples taken at the closest timepoint to 2 weeks prior to calving; Postcalve, Samples collected most immediately following natural calving; and Necropsy, Samples collected from necropsied animals 14–17 weeks postpartum.

More differences in alpha diversity were observed between VT combinations in the vaginal samples (Figure 2D). Unvaccinated Postcalve vaginal communities had significantly lower Shannon indices compared to most other vaginal VT combinations, representing an average ~ 20% decrease in effective species (Table 2). Additionally, more significant differences between VT combinations were identified when comparing Gini-Simpson indices than when comparing the Shannon indices. For this index, Postcalve communities of both vaccination statuses were significantly less diverse (~20% decrease in effective species) when compared to the majority of VT combinations; however, the unvaccinated and vaccinated Postcalve communities were not significantly different from each other in terms of either index. Pairwise comparisons of Gini-Simpson indices also revealed that Precalve and 2W VT combinations had significantly different alpha diversities. In the PCoA analysis, Postcalve samples of both vaccinated and unvaccinated animals cluster together but were more separated from the other timepoints. Additionally, vaginal samples from different timepoints tended to be separated along the first principal coordinate (Figure 3A).

### The elk fecal and vaginal microbiota differ in terms of their most abundant taxa

**Figure 4:**
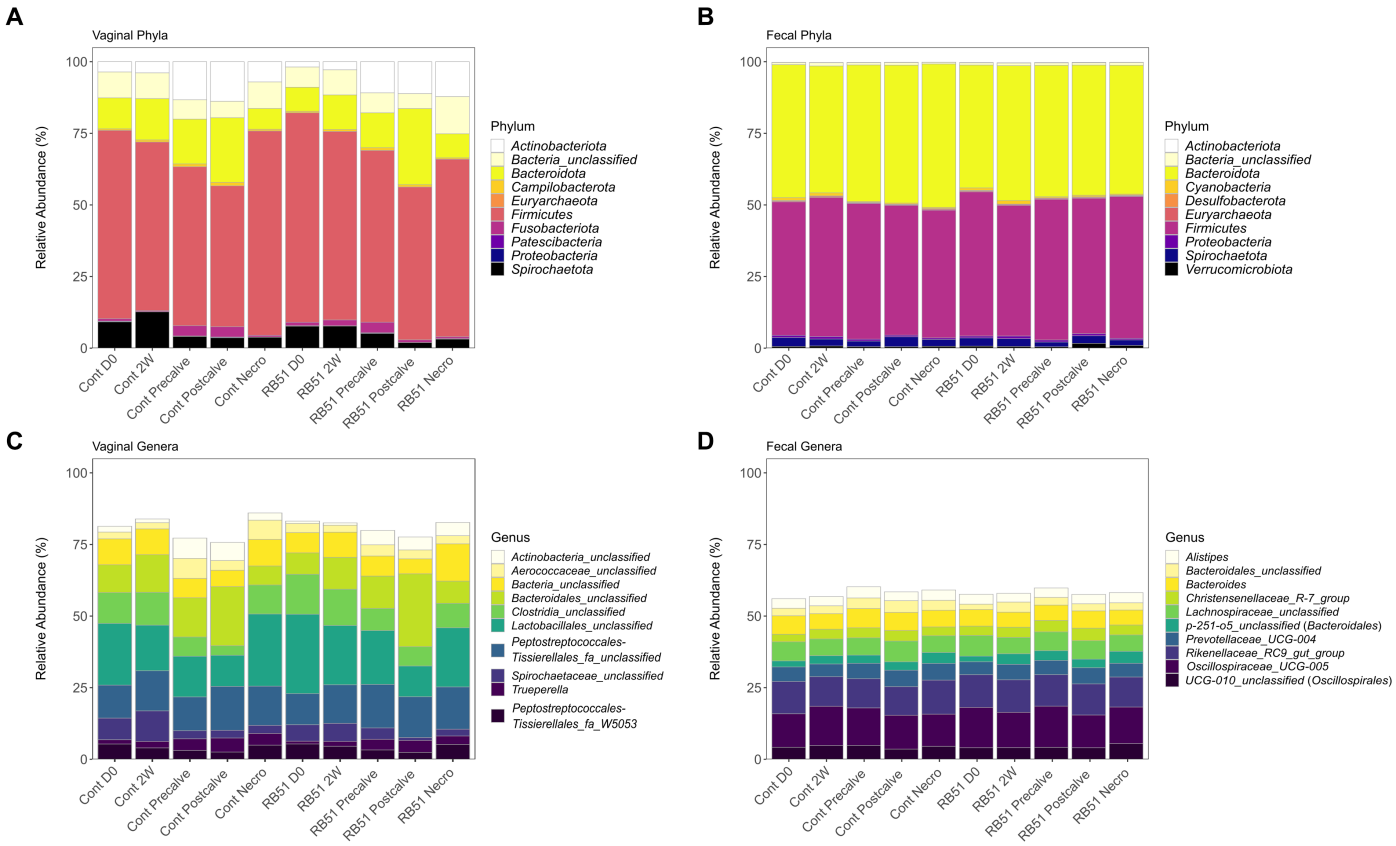
Relative abundance (based on raw reads) of the top 10 most abundant vaginal phyla (panel **A**), fecal phyla (panel **B**), vaginal genera (panel **C**), and fecal genera (panel **D**). Cont, Samples from unvaccinated controls; Necro, Samples taken at Necropsy; and RB51, Samples from RB51-vaccinated animals.

The relative abundance of the top 10 most abundant phyla and genera in the fecal and vaginal datasets are shown in Figure 4. At the phylum level, fecal communities were primarily composed of organisms belonging to *Firmicutes* (~47% relative abundance) and *Bacteroidetes* (~46%) (Figure 4B). *Firmicutes* was also the most dominant phylum in vaginal samples (~62%). *Bacteroidota* in vaginal samples were present at a lower abundance compared to fecal samples (~14%), and vaginal samples were also characterized by the presence of *Actinobacteriota* (~8%) and *Spirochatetota* (~6%) (Figure 4A). The genera *Oscillospiraceae UCG-005* (formerly *Ruminococcaceae UCG-005,* ~13%), the *Rikenellaceae RC9 gut group* (~11%), *Prevotellaceae UCG-004* (~5%), and an unclassified genus belonging to *Lachnospiraceae* (~6%), were some of the most abundant genera in the fecal microbiota samples (Figure 4D). In contrast, three of the top genera in the vaginal microbiota were unclassified genera from unclassified families in the orders *Lactobacillales* (~18%), *Bacteroidales* (~13%), and *Peptostreptococcales-Tissierales* (~13%) (Figure 4C). In fecal samples, two of the three most abundant OTUs, OTUs 7 and 21, were classified as *Oscillospiraceae UCG-005* (~5 and ~ 3%, respectively). The third was classified as part of a genus belonging to *p-251-05*, a family of order *Bacteroidales* (OTU 17; ~3%) (Supplementary File 1). The most abundant OTUs in the vaginal samples were unclassified members of the orders *Lactobacillales* (OTU 1; ~9%), *Peptostreptococcales-Tissierellales* (OTU 2; ~7%), and *Bacteroidales* (OTU 3; ~6%). BLAST analysis classified those OTUs as closest to *Suicoccus acidiformans* (OTU 1, ~97% percent identity), *Anaerosphaera aminiphila* (~93% percent identity, OTU 2), and *Porphyromonas macacae* (~91% percent identity, OTU 3), respectively (Supplementary File 1). Family-level relative abundance in the fecal and vaginal microbiota can be found in Supplementary File 1 and Supplementary Figure 2.

### Certain fecal and vaginal taxa are differentially abundant due to vaccination status, timepoint, and the interaction of vaccination status and timepoint

In both the fecal and vaginal microbiota, analysis revealed differentially abundant phyla, genera, and OTUs across vaccination status, timepoint, and the interaction effect between the two. Table 3 lists the total number of differentially abundant taxa per taxonomic level and microbial community as well as the number of differentially abundant taxa driven by a given variable. A full list of all differentially abundant taxa, their false discovery rate, and variable of effect for the fecal and vaginal samples are available in Supplementary Files 2, 3, respectively. For a given source (fecal or vaginal samples) and taxonomic level (phylum, genus, OTU), differential abundance in terms of absolute of log_2_ fold change is shown as a bar chart for all differentially abundant taxa (Supplementary Figures 3–8). Within a given supplementary figure, differentially abundant taxa are grouped by variable of effect (timepoint, vaccination, or interaction effect).

**Table 3:** Number of differentially abundant (DA) taxa per variable.

	Total number unique DA taxa	Affected by timepoint	Affected by vaccination status	Affected by interaction effect	Affected by time and vaccination but no interaction
Fecal phyla	11	8	3	0	0
Fecal genera	55	51	8	0	4
Fecal OTUs	44	34	5	7	2
Vaginal phyla	8	5	0	3	0
Vaginal genera	72	41	5	29	3
Vaginal OTUs	127	75	8	48	4

### Reads belonging to the *Brucella abortus* 16S rRNA gene were not found in the dataset of Han et al. (2023)

BLAST analysis of the aforementioned dataset did not identify any reads with more than ~95% nucleotide identity to the 16S rRNA gene of *B. abortus* 2308, below the 98% nucleotide identity threshold (32). The BLAST results with the highest scores can be found in Supplementary File 4.

## Discussion

*Brucella abortus* both causes economic and veterinary losses in agriculture and poses a threat to human health as a chronic disease. Despite eradication efforts, *B. abortus* remains endemic to the GYA where elk serve as an important wildlife reservoir and transmission risk to domestic cattle. Understanding the elk immune responses to vaccination and challenge are thus critical for the development of improved vaccination approaches against brucellosis. Elk display a distinct response to *B. abortus* vaccination and infection compared to cattle and bison, potentially due in part to interspecific differences in immunology (15, 19, 23, 25). However, the mechanisms behind this phenomenon remain incompletely understood. As part of efforts to further understand the elk host response to *B. abortus*, we were interested in the microbial communities of elk challenged with *B. abortus* as well as the effect of RB51 vaccination on the elk microflora. Therefore, we conducted 16S rRNA gene amplicon sequencing to compare the fecal and vaginal microbiota of periparturient RB51-vaccinated and non-vaccinated elk in response to *B. abortus* challenge.

### *Brucella abortus* challenge

All unvaccinated and vaccinated elk in this study experienced normal pregnancies and gave birth to live calves, with the exception of one cow and calf lost to dystocia. While previous research has shown that the rate of abortion in elk due to brucellosis tends to be lower as compared to cattle or bison (15, 21–26), we did not observe any abortions in this study, which was unexpected. However, *B. abortus* 2308 was successfully cultured from the tissues of all but two elk at necropsy, indicating that conjunctival infection and subsequent dissemination occurred. Further, all animals seroconverted positive to the *B. abortus* 2308 strain by 4 weeks post challenge (data not shown), indicating that immune responses to infection did occur which suggests a successful challenge. There is therefore no obvious cause, methodological or otherwise, that explains the absence of abortion.

With the exception of OTU 43 which was flagged as a contaminant (decontam *p* score = 0.004), we did not observe any *Brucella* species in the vaginal or fecal microbiota. The absence of *B. abortus* at the gestation timepoints (D0, 2W, Precalve) is consistent with what is known about *Brucella* shedding (6). However, somewhat surprisingly, we did not detect any *Brucella* at the Postcalve or Necropsy timepoints. The absence of detectable *B. abortus* may be explained by two factors. First, even though elk were successfully infected, the lack of abortions suggests that localization to and infection of placentomes by *B. abortus* may not have occurred. Without localization of the bacteria to the placenta, we would not expect shedding of the pathogen in the vaginal tract after calving. Alternatively, *B. abortus* could have localized to those tissues, but the sampling window may have been too late to detect shedding bacteria. This could be due to the absence of preferred host tissues to colonize following parturition, specifically the placental trophoblasts for which *B. abortus* exhibits a strong tropism. A study using similar conjunctival challenge in elk supports the idea that sampling times may have missed *B. abortus* shedding; in this case, *B. abortus* 2308 was culturable by vaginal swab only within a few days after an abortion event, although a small minority of cows were culture-positive for up to ~4 weeks (23). *Brucella abortus* culture data from elk vaginal exudates further indicates that the window of detection is narrower for normal parturitions than for abortions or delivery of nonviable calves (26). Therefore, it is not unreasonable that we did not detect *B. abortus* in the vaginal samples as no animals experienced abortions and the Postcalve timepoint was ~2 weeks after parturition. Our analysis also did not detect any *Brucella* in the fecal microbiota. Consistent with this finding, *B. abortus* was not isolated from the feces of elk cows challenged with *B. abortus* (23) or from the feces of viable, seropositive elk calves born to infected cows (26). Similarly, Han et al. (32) did not report *B. abortus* in the fecal microbiota of mice infected orally with *B. abortus* 2308; our BLAST analysis of the entire Han et al. dataset also did not identify reads with high percent nucleotide identity to *B. abortus*. Importantly, the authors were able to recover *B. abortus* from intestinal and immune tissues, suggesting that in some circumstances *B. abortus* is not actively shed luminally even if it is culturable from neighboring tissues (32). Future experiments could determine if and when *B. abortus* is detectable in fecal and vaginal samples by 16S rRNA gene amplicon sequencing by performing longitudinal sampling on a finer scale.

### General observations of the elk fecal and vaginal microbiota

The composition of the elk fecal microbiota agrees with previous studies of elk and other ruminants. The dominant phyla in the elk feces were *Firmicutes* and *Bacteroidota*, both of which have been previously observed in the feces, rumen, and lower gastrointestinal tract of elk (47–51). These phyla are commonly the predominant phyla in ruminants (52, 53), with the ratio of *Firmicutes* to *Bacteroidota* being implicated in animal health and growth (54–58). At the genus level, we observed high abundances of genera including *Oscillospiraceae UCG-005*, the *Rikenellaceae* RC9 gut group, and *Prevotellaceae UCG-004* as well as an uncultured member of *Lachnospiraceae*. These taxa are common to the gastrointestinal microbiota of ruminants including elk (47, 49, 51, 52, 59–62). Notably, the *Rikenellaceae* RC9 gut group is known to produce a variety of short chain fatty acids which are then utilized by the ruminant host (59, 60), and members of *Lachnospiraceae* are implicated in butyrate production, milk-fat yield, and feed efficiency in ruminants (52, 54, 63). Similarly, *Oscillospiraceae UCG-005* is positively correlated with isobutyrate production (64). The above taxa have also been identified in similar proportions in other members of the genus *Cervus* including *C. nippon* (sika deer) and *C. elaphus* (red deer) (65–69). Therefore, the composition of the elk fecal microbiota in this study corroborates previous analyses of North American elk and other ruminants.

Although the gastrointestinal and fecal microbiota of members of the genus *Cervus* have been the focus of multiple analyses, to our knowledge no analysis of the vaginal microbiota has been conducted for any *Cervus* species. Here, we observed that the vaginal communities were strongly distinct in composition from the fecal microbiota samples. This work demonstrates that *Firmicutes* dominated the elk vaginal microbial community with a mixture of *Bacteroidota*, *Actinobacteriota*, and *Spirochaetota* making up the remaining phyla which had relative abundances greater than 2%. The above phyla have all been identified in the vaginal microflora of cattle and sheep (70–72). However, the relative abundance of *Bacteroidota* tends to be higher in cattle vaginal samples than what we observed in elk. *Proteobacteria* were even less prevalent in the elk vaginal samples (relative abundance <1%) than what has been documented for both cattle and sheep (70, 72–75). The three most abundant genera in the elk vaginal microbiota belonged to unclassified families belonging to the orders *Lactobacillales*, *Bacteroidales*, and *Peptostreptococcales-Tissierellales*. These orders all contain taxa previously documented in ruminant vaginal tracts (71, 76, 77). BLAST analysis indicated that OTUs 1, 2, and 3 were most similar to organisms in the genera *Suicoccus*, *Anaerosphaera*, and *Porphyromonas*, respectively. *Suicoccus* is a recently formed, monotypic genus containing the species *S. acidiformans* which was isolated from lymph nodes of diseased pigs (78), and it belongs to the same family as *Facklamia* and *Anaerocococcus* which have previously been determined to be members of the ruminant vaginal microflora (72, 74, 79, 80). *Anaerosphaera* has previously been identified in the vaginal tract of pigs but has also been identified in human feces and in a bioreactor (81–83). Finally, the genus *Porphyromonas* has been documented in multiple ruminant vaginal studies (71, 72, 75). While this genus can be found among the flora of the healthy bovine reproductive tract (74, 75, 84), multiple studies have found this genus to be positively correlated with cattle reproductive diseases including metritis, necrotic vulvovaginitis, and repeat breeder syndrome (73, 76, 85–91). Notably, *Porphyromonas levii* is capable of invading cattle endometrial tissues and also produces a protease that cleaves bovine IgG, demonstrating that it possesses adaptations to a pathogenic lifestyle (92, 93). It is possible then that *Porphyromonas* species may also be opportunistic reproductive tract pathogens in elk.

It should be noted that although D0 timepoint samples give a profile of healthy elk, all other samples were from animals infected with *B. abortus* 2308. Therefore, the aggregate microbial communities described above are unlikely to be completely representative of the fecal and vaginal microbiota of healthy periparturient elk. Furthermore, animal care conditions would alter the microbiota from what would be expected of wild elk. However, as a whole, these results strongly agree with the existing paradigms of elk and ruminant gastrointestinal and urogenital microbial ecology.

### The elk vaginal microbial community structure is more affected by time and vaccination

In this study, we identified significant differences in alpha diversity between VT combinations within the vaginal and fecal datasets. More of these differences were in Gini-Simpson index than in Shannon index; because the Shannon index emphasizes the richness of a community, whereas the Gini-Simpson index emphasizes the evenness, it can be broadly stated that most of the significantly different VT combinations differed in the evenness of their microbial communities rather than in richness (94, 95).

There were fewer significant differences between fecal VT combinations; additionally, fecal VT combinations clustered together in the PCoA plots. Taken together, this suggests that neither time nor vaccination status were responsible for major shifts in the elk fecal microbiota on the community structure scale. Previous work indicates that *B. abortus* oral infection causes shifts in fecal microbiota alpha diversity over time (32). Notably elk in this study were infected with *B. abortus* 2308 via the conjunctival rather than the oral route. Still, the absence of an obvious effect of time on fecal microbiota alpha diversity was somewhat unexpected as research indicates that the fecal microbiota community structure shifts during the periparturient period in both cattle and swine in part due to shifts in host physiology and metabolism (96–101). One potential explanation for this observation is the feeding of a single diet to elk in our study over the course of the sampling period. Contrasting the methods used in this work, all the aforementioned studies except Sun et al. switched the diet of the animals over the course of the periparturient period (in most cases immediately after parturition). This was necessary for animal health and reflects conventional agriculture practice, but it results in confounding of dietary changes with time. Therefore, it is possible that previous observations of community-scale shifts in fecal microbiota over parturition are primarily due to diet rather than physiological changes in the host. Regarding the effects of vaccination on fecal alpha diversity, a number of studies agree with our observation that vaccination does not affect fecal microbiota alpha diversity. These included research on vaccination of Rhesus macaques against HIV-1 (102), mice with Bacillus Calmette-Guérin (BCG) (103), striped skunk against rabies (104), and foals against *Rhodococcus equi* (105). In some cases, however, vaccination can alter fecal community structure as observed in a study of piglets vaccinated against *Lawsonia intraceullularis*, a gastrointestinal pathogen (106).

Compared to fecal samples, more differences in VT combination alpha diversity were present for the vaginal samples, indicating that the vaginal microbiota community structure was more affected by the study conditions. Notably, the control-Postcalve community possessed a significantly lower Shannon and Gini-Simpson index compared to the majority of VT combinations; this was also the case for the RB51-Postcalve microbiota. Both these decreases in Shannon and Gini-Simpson index reflected an approximately 20% decrease in the number of effective species in the vaginal microbiota at Postcalve compared to the other timepoints. It is well-established in the literature that parturition induces a significant, discrete shift in microbial community structure and composition in the mammalian reproductive tract (76, 84, 107–110). In particular, Bicalho et al. and Kudo et al. also observed a trend of decrease in alpha diversity in cattle vaginal microbiota following parturition (84, 110). The mammalian reproductive tract alpha diversity is also known to shift longitudinally during pregnancy but prior to parturition, corroborating the additional differences between VT combinations of different timepoints as well as the PCoA plots (72, 111–114). Vaccination status did not affect vaginal alpha diversity as measured by either index. This could be a reflection of reduced efficiency of vaccination in elk, in which case fewer differences would be expected between RB51-vaccinated and unvaccinated animals. Alternatively, vaccination might not greatly alter vaginal communities as suggested by research in cattle which observed no differences in vaginal community structure in response to any of three metritis vaccines (115). Similarly, HIV-1 vaccination did not affect vaginal alpha diversity in Rhesus macaques (102). Collectively, the data suggest that time but not vaccination status was the driver of shifts in vaginal microbiota community structure in this study. It is however possible that community structure shifts occurred in the elk fecal and/or vaginal microbiota soon after vaccination but that the community had reverted to its original structure at the time of sampling, but additional experiments would be necessary to confirm this.

### Time and vaccination status affect the relative abundance of individual fecal and vaginal taxa

While timecourse through challenge and parturition did not greatly affect the elk fecal community structure, it was the driving variable behind the majority of differentially abundant fecal taxa. Among these were highly abundant taxa including the phyla *Bacteroidetes*, *Firmicutes*, and *Spirochaetota* and genera including *Oscillospiraceae UCG-005* and the *Rikenellaceae* RC9 gut group. *Spirochaetota* significantly increased in relative abundance from Precalve to Postcalve while the other taxa significantly decreased over the same interval. Muñoz-Vargas et al. similarly observed a decrease in *Firmicutes* and *Oscillospiraceae* and an increase in *Spirochaetota* in dairy cattle over periparturition, but they did not observe a significant change in *Bacteroidetes* or *Rikenellaceae* (98). In the cattle rumen microbiota, Pitta et al. observed that *Firmicutes, Spirochaetes*, and *Oscillospiraceae* decreased in relative abundance over parturition while *Bacteroidetes* increased (116); in another study by Zhu et al., *Bacteroidetes* was found to decrease along with *Spirochaetes* and *Ruminococcaceae* in the rumen over parturition while *Firmicutes* were unaffected in terms of relative abundance (117). Importantly, animals in the first two aforementioned studies received different diets after calving whereas a consistent diet was used in the study by Zhu et al., potentially contributing to some of the differences in differentially abundant taxa between the studies. Therefore, interpreting the results of this work in elk should be done with the use of a consistent diet in mind. However, because the rumen microbiome and fecal microbiome are unique environments, results from one do not necessarily translate to the other. Regardless, we can confidently conclude that the course of pregnancy and *B. abortus* infection affected the relative abundance of multiple fecal taxa, often in ways comparable to what has been observed in the microbiomes of other ruminants.

Time was also the primary factor driving differentially abundant taxa in the elk vaginal microbiota. One of the more interesting patterns we observed was the temporal dynamics of known vaginal pathogens associated with metritis in other ruminants. We observed that OTUs 3 and 5 (classified by BLAST as *Porphyromonas*) as well as OTU 9 (*Trueperella*) increased in relative abundance from Precalve to Postcalve in agreement with the literature. Their parent genera increased over this interval as well (84, 85, 110, 118). As discussed previously, *Porphyromonas* can colonize and invade ruminant uterine tissues and is associated with a number of reproductive tract diseases. The genus *Trueperella* and particularly the species *T. pyogenes* are often associated with metritis and purulent vaginal discharge in cattle; indeed, *T. pyogenes* is capable of causing a number of pathologies of the reproductive tract tissues in ruminants (71, 84, 90, 91, 119). However, the *Trueperella* isolate most abundant in this study (OTU 9) had less percent identity to *T. pyogenes* than to *T. bialowiezensis*, a proposed etiological agent of a necrotic disease of the penis of European bison (120, 121). Outside of its implications in disease in bison the ecology, distribution, and lifestyle of *T. bialowiezensis* are unknown. This, combined with the higher relative abundance of *Trueperella* (~3%) observed here compared to prior studies suggests that the biological relevance of *Trueperella* in the elk reproductive tract is different than in that of cattle (84, 122). Finally, OTU 22 was classified as *Helcococcus* and also increased in abundance from Precalve to Postcalve. *Helcococcus* is implicated in metritis in cattle where it appears to synergize with other reproductive pathogens including *Trueperella* and *Fusobacterium* (76, 85, 86, 88, 118). Overall, our results agree with existing literature that many taxa containing known reproductive pathogens increase in abundance following the major perturbation of the ruminant vaginal microbiota caused by parturition. Under normal circumstances, these pathogens are eventually suppressed and the microbiota returns to homeostasis, but reproductive disease can result if these pathogens overcome the intrinsic and extrinsic factors of the microbiome that keep them in check. In this study, no obvious clinical signs of reproductive disease were observed in the elk cows following parturition.

In comparison to time, vaccination status and the interaction of time and vaccination status affected far fewer taxa in the fecal microbiota. Normal but less abundant flora of the ruminant GI tract were differentially abundant in response to vaccination status including OTU 126 (*M2PB4–65 termite gut group*) and the genus *Bacteroidales UCG-001* (60–62). It is possible that changes in the relative abundance of fecal taxa may be indicative of changes in the relative abundance of rumen taxa, but direct analysis of the rumen microbiome would be necessary to confirm this. This would be an intriguing avenue of research, as vaccine-induced shifts in rumen microbial taxa could be accompanied by shifts in host physiology and metabolism. Interestingly, the phylum *Fusobacteriota* was more abundant in the feces of unvaccinated elk. One species of this phylum, *Fusobacterium necrophorum*, is a notorious bovine reproductive tract pathogen associated with metritis (71, 85, 86, 88, 91). Additionally, Koester et al. (72) observed that *Fusobacterium* was enriched in ewes which did not establish pregnancy, indicating that *Fusobacterium* can potentially prevent pregnancy in addition to causing reproductive lesions postpartum. Interestingly, Tasara et al. (91) also observed that *Fusobacteriota* was significantly more abundant in the feces of cattle with metritis, potentially indicating an association with *Fusobacteriota* in the gastrointestinal tract with reproductive disease. However, healthy cattle are known to contain *Fusobacteriota* within the rumen epithelial microbiome (123). Given the potential for fecal matter to contaminate the female reproductive tract in ruminants, this observation of reproductive pathogens in the feces may be relevant for animal health.

The differential abundance of potential reproductive tract pathogens across vaccination status was even more apparent in the elk vaginal microbiota. Compared to time, more differentially abundant taxa were present in the vaginal microbiota as a result of vaccination status and the interaction of vaccination and time. Many of these were known reproductive tract pathogens in ruminants. Two OTUs classified by BLAST as *Porphyromonas*, 15 and 56, were differentially abundant according to vaccination status and the interaction effect, respectively. OTU 15 was significantly more abundant in vaccinated elk while OTU 56 was significantly more abundant in unvaccinated elk at D0, 2W, and Precalve. OTU 81, classified as an unknown bacterium, was also differentially abundant due to vaccination status alone and was more prevalent in unvaccinated animals. BLAST found that the representative sequence of OTU 81 was most homologous to members of class *Mollicutes* with 89% similarity to *Mycoplasma feliminutum*. OTU 150 was classified similarly and was more abundant in RB51-vaccinated elk at Postcalve and Necropsy. While *M. feliminutum* is typically associated with felines, it has been observed previously in bovine skin lesions (124), and other members of the genus *Mycoplasma* are associated with bovine reproductive disease (89, 125). Finally, OTU 316 was classified as *Fusobacterium necrophorum* by BLAST analysis and was significantly more abundant in vaccinated elk at Precalve. Unvaccinated elk also had significantly higher abundances of another *Fusobacterium*, OTU 1437, than vaccinated elk; however, OTU 1437 only had an overall relative abundance of ~0.01%. The effect of vaccination status on so many putative reproductive pathogens was unexpected, and there was no clear trend between vaccination status and the abundance of putative reproductive pathogens. In some cases, such as for the *Porphyromonas*, *Mycoplasma*, and *Fusobacterium* OTUs mentioned above, different OTUs belonging to a single genus had opposite correlations with vaccination status. This broad shift in relative abundance of reproductive pathogens was not observed in the vaginal microbiota of cattle administered metritis vaccines targeting *E. coli* and *F. necrophorum*. The authors did observe a decrease in *F. necrophorum* and the reproductive pathogen *Bacteroides heparinolyticus* in vaccinated animals at day 9 postpartum, but other pathogens such as *Mycoplasma*, *Helcococcus*, and *Porphyromonas* were unaffected by vaccination status (115). Therefore, additional work is necessary to determine how common shifts in reproductive pathogen relative abundance is in response to vaccination.

Although shifts in metabolism and host physiology during periparturition likely explain the majority of differentially abundant taxa driven by time, the mechanism by which vaccination affected the elk fecal and vaginal microbiota is less clear. These shifts in microbiota occurred even prior to challenge with virulent *B. abortus* as evidenced by differences between vaccinated and unvaccinated animals at D0. In agreement with this study, prior investigations of the effects of HIV-1 vaccination in Macaques and SARS-CoV-2 vaccination in humans demonstrated a shift in the relative abundance of multiple gut taxa before and after vaccination (102, 126). Similarly, differences in relative abundance of taxa were observed in the respiratory and gut microbiota between BCG-vaccinated and unvaccinated mice and in the vaginal microbiota between control cattle and cattle vaccinated against metritis pathogens (103, 115). Therefore, it appears that vaccination affects the relative abundance of certain microbes perhaps by altering the host immune response and physiology. Indeed, there exists a complex web of interplay between resident microflora, the host innate and adaptive immune systems, and foreign and opportunistic pathogens, and this interplay has important implications in both the microbiome and in vaccine efficacy (27–30). In Rhesus macaques, for example, it was observed that rectal *Prevotella* relative abundance was negatively correlated with the number of gut-homing CD4 T cells at the same timepoint; the same study also showed that *Prevotella* relative abundance inversely correlated with IgG production against HIV-1 16 weeks after HIV-1 vaccination (102). This literature suggests that the differences in microbiota between vaccinated and unvaccinated elk are likely due to changes in the elk immune system. Because RB51 is a live-attenuated vaccine which disseminates from the conjunctival route, it is also possible that a localized immune response in the reproductive tract against RB51 had additional effects on local microflora (18).

In the future, replicating this study with the addition of uninfected elk would help distinguish between effects due to the interaction of vaccination and *B. abortus* infection and effects solely due to vaccination or *B. abortus* challenge alone. Such an experiment would allow further exploration of the effects of RB51 vaccination and *B. abortus* infection on the vaginal microbiota and any potential implications on the health of the reproductive tract. Based on the results presented here, RB51 vaccination is capable of altering the relative abundance of known reproductive tract pathogens, but the relevance of this phenomenon to animal health requires further study. Our methodology could also be applied to an analysis of cattle or bison microbiomes. Because these animals are relatively more susceptible to *B. abortus*, a comparative analysis of their microbiota could potentially identify unique and shared taxa among ruminants that play a role in *B. abortus* infection. For example, correlating brucellosis outcome in each species with microbial taxa could be used to identify bacteria that synergize with *B. abortus* as well as bacteria which compete with or suppress *B. abortus* infection. There is therefore the possibility that differences in microbial taxa between elk, cattle, and bison are correlated with the level of susceptibility to *B. abortus* and subsequent brucellosis.

## Conclusion

We conducted a longitudinal 16S rRNA gene amplicon sequencing study of the fecal and vaginal microbiota of unvaccinated and RB51-vaccinated pregnant elk challenged with *B. abortus* 2308. In addition to being the first study of the vaginal microbiota of an animal infected with *B. abortus*, this study is also the first to analyze the vaginal microbiota of any *Cervus* species. The results demonstrated that the fecal and vaginal microbiota of elk were similar in composition to that of other ruminants; additionally, the longitudinal shifts in both fecal and vaginal taxa over parturition similarly agree with the literature. Notably, we observed differences in community structure and taxa abundance in both sample types between vaccinated and unvaccinated elk even before *B. abortus* challenge. Among the differentially abundant taxa in the vaginal communities, multiple genera were associated with reproductive tract disease in cattle. Many of these taxa increased in abundance postpartum, highlighting how parturition disrupts the ruminant vaginal microbiome and increases vulnerability to opportunistic pathogens. Thus, RB51 vaccination is associated with differences in the elk fecal and vaginal microbiota, and this phenomenon is potentially relevant to animal health.

## Data availability statement

Raw sequencing data for this project are submitted to the SRA under BioProject ID PRJNA1031606. Code used for the analysis can be found at https://github.com/benc347?tab=repositories.

## Ethics statement

The animal study was approved by National Animal Disease Center Animal Care and Use Committee. The study was conducted in accordance with the local legislation and institutional requirements.

## Author contributions

BT-C: Investigation, Methodology, Writing – original draft, Writing – review & editing, Formal analysis, Validation, Visualization. FR-S: Formal analysis, Investigation, Methodology, Writing – review & editing. SS-E: Methodology, Writing – review & editing, Conceptualization, Data curation, Supervision. PB: Conceptualization, Data curation, Methodology, Writing – review & editing. SO: Conceptualization, Data curation, Methodology, Writing – review & editing, Supervision. EP: Conceptualization, Data curation, Methodology, Supervision, Writing – review & editing, Investigation, Writing – original draft.

## Footnotes

^1^ https://earthmicrobiome.org/protocols-and-standards/16s/

^2^ https://mothur.org/wiki/silva_reference_files/

^3^ https://github.com/mooreryan/divnet-rs

^4^ https://blast.ncbi.nlm.nih.gov/blast/Blast.cgi?PAGE=Nucleotides&PROGRAM=blastn&PAGE_TYPE=BlastSearch&BLAST_SPEC=SRA

## Supplementary material

SUPPLEMENTARY FIGURE 1 Venn diagram showing the number of OTUs which possess a relative abundance of greater than 0.01% in each community of interest (unvaccinated fecal, vaccinated fecal, unvaccinated vaginal, vaccinated vaginal). The total number of OTUs with >0.01% relative abundance is listed by the community label.

SUPPLEMENTARY FIGURE 2 Relative abundance (based on raw reads) of the top 10 most abundant families for each sample type. Abbreviations: Cont – samples from unvaccinated controls. Necro – samples taken at Necropsy. RB51 – samples from RB51-vaccinated animals.

SUPPLEMENTARY FIGURE 3 Fecal phyla which were differentially abundant due to time, vaccination status, or the interaction of time and vaccination status. Differentially abundant taxa are separated by variable of effect (timepoint, vaccination, or interaction effect), and differences in abundance are displayed in absolute log_2_fold change.

SUPPLEMENTARY FIGURE 4 Fecal genera which were differentially abundant due to time, vaccination status, or the interaction of time and vaccination status. Differentially abundant taxa are separated by variable of effect (timepoint, vaccination, or interaction effect), and differences in abundance are displayed in absolute log_2_fold change.

SUPPLEMENTARY FIGURE 5 Fecal OTUs which were differentially abundant due to time, vaccination status, or the interaction of time and vaccination status. Differentially abundant taxa are separated by variable of effect (timepoint, vaccination, or interaction effect), and differences in abundance are displayed in absolute log_2_fold change.

SUPPLEMENTARY FIGURE 6 Vaginal phyla which were differentially abundant due to time, vaccination status, or the interaction of time and vaccination status. Differentially abundant taxa are separated by variable of effect (timepoint, vaccination, or interaction effect), and differences in abundance are displayed in absolute log_2_fold change.

SUPPLEMENTARY FIGURE 7 Vaginal genera which were differentially abundant due to time, vaccination status, or the interaction of time and vaccination status. Differentially abundant taxa are separated by variable of effect (timepoint, vaccination, or interaction effect), and differences in abundance are displayed in absolute log_2_fold change.

SUPPLEMENTARY FIGURE 8 Vaginal OTUs which were differentially abundant due to time, vaccination status, or the interaction of time and vaccination status. Differentially abundant taxa are separated by variable of effect (timepoint, vaccination, or interaction effect), and differences in abundance are displayed in absolute log_2_fold change.
