# Corrigendum: Fecal and vaginal microbiota of vaccinated and non-vaccinated pregnant elk challenged with *Brucella abortus*

**DOI:** 10.3389/fvets.2024.1409301

**Published:** 2024-04-30

**Authors:** Bienvenido W. Tibbs-Cortes, Faith M. Rahic-Seggerman, Stephan Schmitz-Esser, Paola M. Boggiatto, Steven Olsen, Ellie J. Putz

**Affiliations:** ^1^Infectious Bacterial Diseases Research Unit, United States Department of Agriculture, Ames, IA, United States; ^2^Interdepartmental Microbiology Graduate Program, Iowa State University, Ames, IA, United States; ^3^Department of Animal Science, Iowa State University, Ames, IA, United States

**Keywords:** brucellosis, microbiome, elk, RB51, pregnancy, fecal, vaginal

In the published article, the acknowledgements section was mistakenly not included in the publication. The missing section appears below:

The authors apologize for this error and state that this does not change the scientific conclusions of the article in any way. The original article has been updated.

