# Supplementary figures and images for: Fecal and vaginal microbiota of vaccinated and non-vaccinated pregnant elk challenged with Brucella abortus
Source: Front Vet Sci. 2024 Jan 30;11:1334858. doi: 10.3389/fvets.2024.1334858 (PMC10861794; doi:10.3389/fvets.2024.1334858)

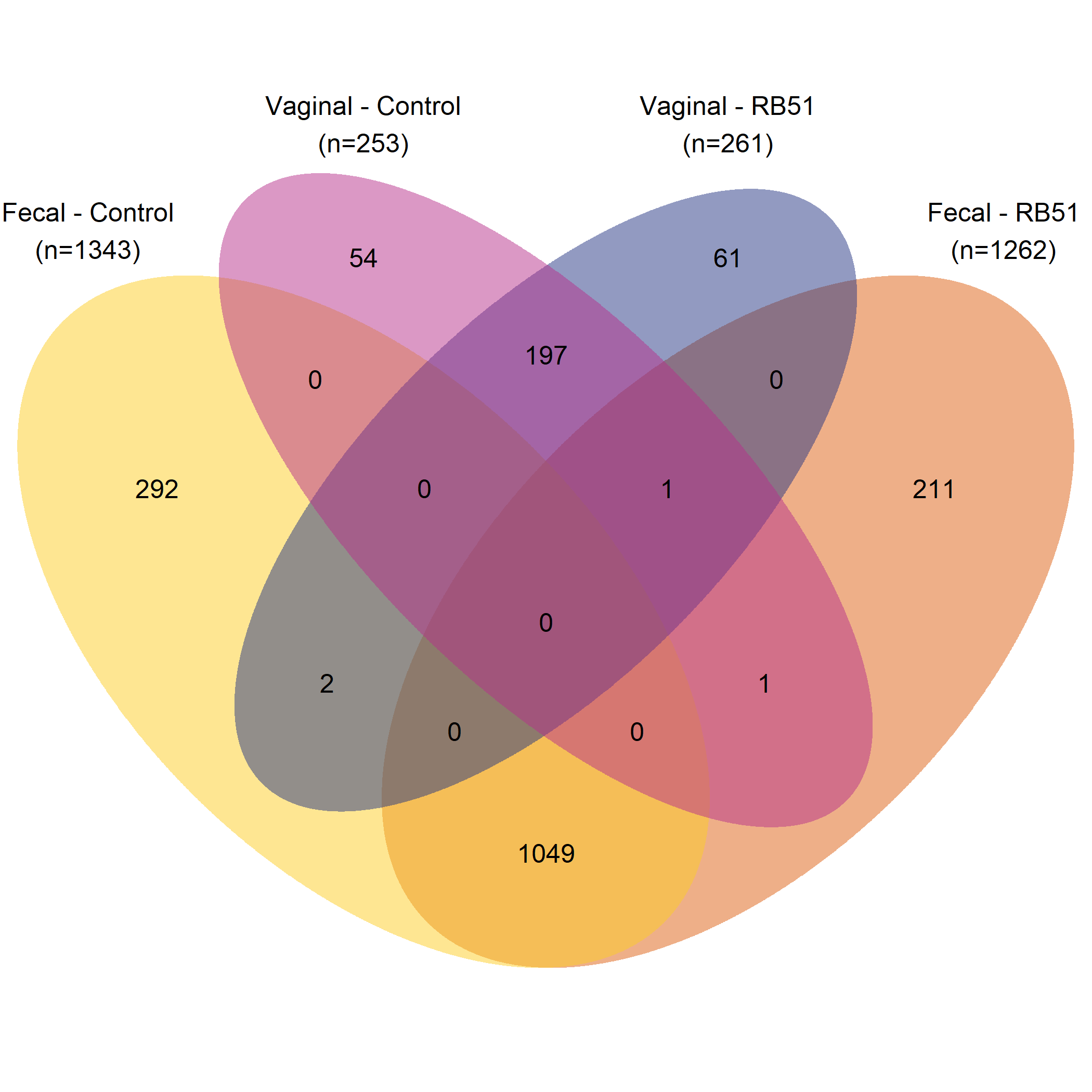

Supplement: SUPPLEMENTARY FIGURE 1 — Venn diagram showing the number of OTUs which possess a relative abundance of greater than 0.01% in each community of interest (unvaccinated fecal, vaccinated fecal, unvaccinated vaginal, vaccinated vaginal). The total number of OTUs with >0.01% relative abundance is listed by the community label. [file Image_1.png]

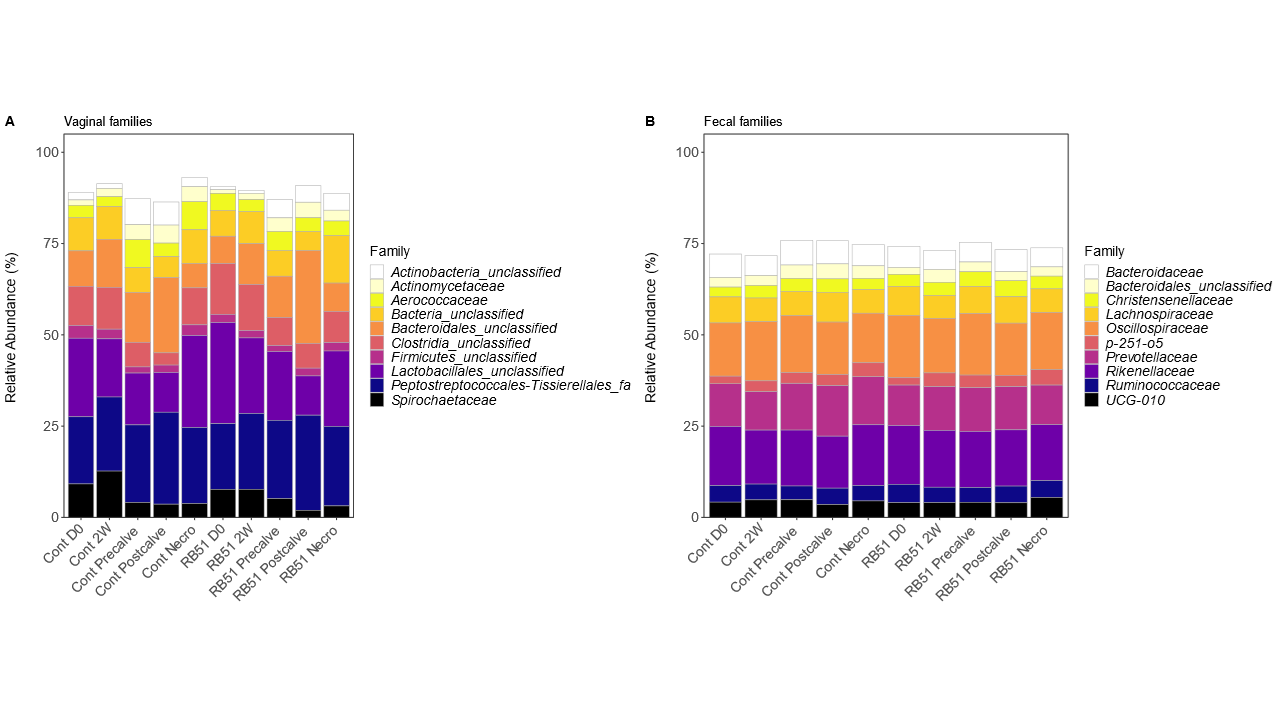

Supplement: SUPPLEMENTARY FIGURE 2 — Relative abundance (based on raw reads) of the top 10 most abundant families for each sample type. Abbreviations: Cont – samples from unvaccinated controls. Necro – samples taken at Necropsy. RB51 – samples from RB51-vaccinated animals. [file Image_2.TIF]

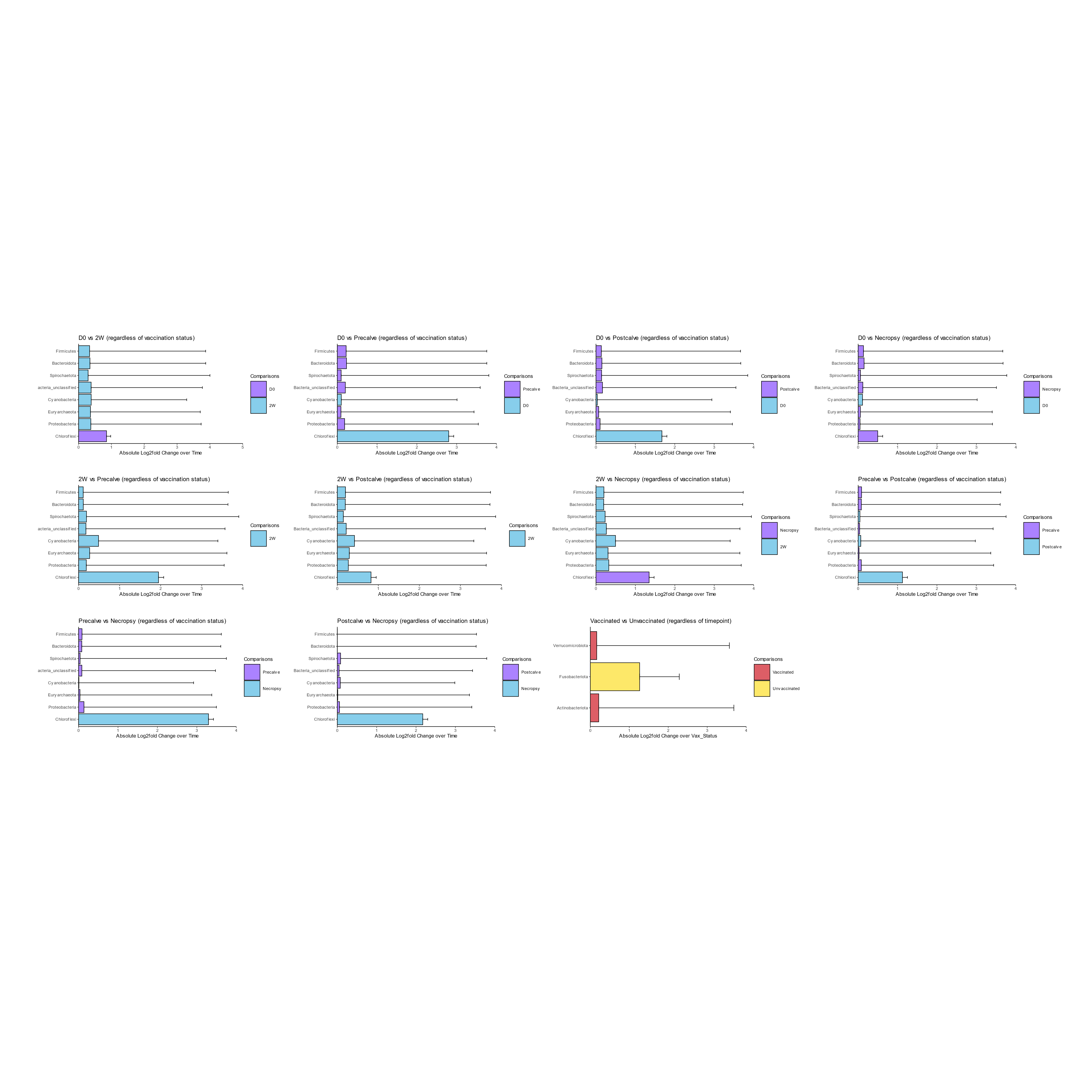

Supplement: SUPPLEMENTARY FIGURE 3 — Fecal phyla which were differentially abundant due to time, vaccination status, or the interaction of time and vaccination status. Differentially abundant taxa are separated by variable of effect (timepoint, vaccination, or interaction effect), and differences in abundance are displayed in absolute log2fold change. [file Image_3.TIF]

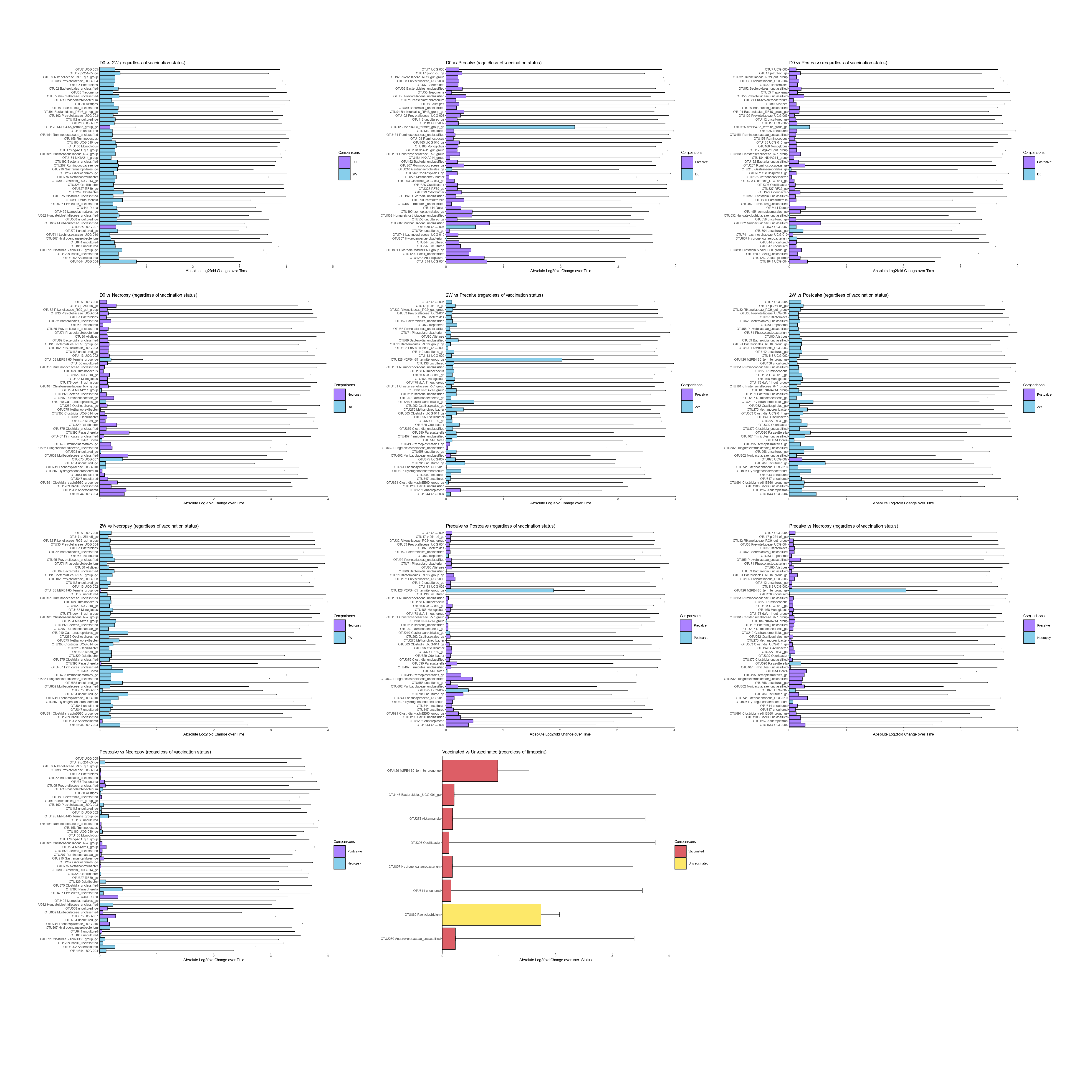

Supplement: SUPPLEMENTARY FIGURE 4 — Fecal genera which were differentially abundant due to time, vaccination status, or the interaction of time and vaccination status. Differentially abundant taxa are separated by variable of effect (timepoint, vaccination, or interaction effect), and differences in abundance are displayed in absolute log2fold change. [file Image_4.TIF]

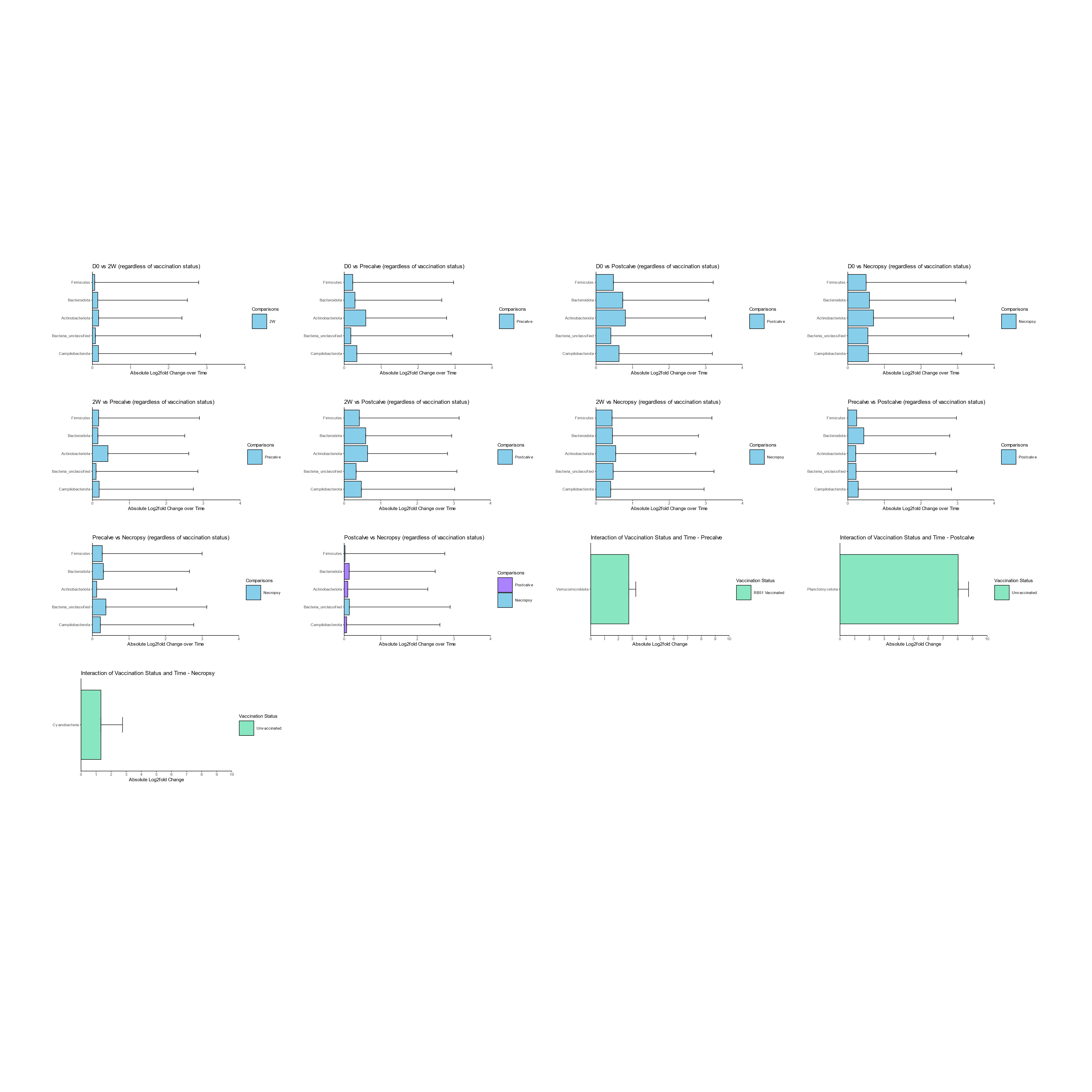

Supplement: SUPPLEMENTARY FIGURE 6 — Vaginal phyla which were differentially abundant due to time, vaccination status, or the interaction of time and vaccination status. Differentially abundant taxa are separated by variable of effect (timepoint, vaccination, or interaction effect), and differences in abundance are displayed in absolute log2fold change. [file Image_6.TIF]

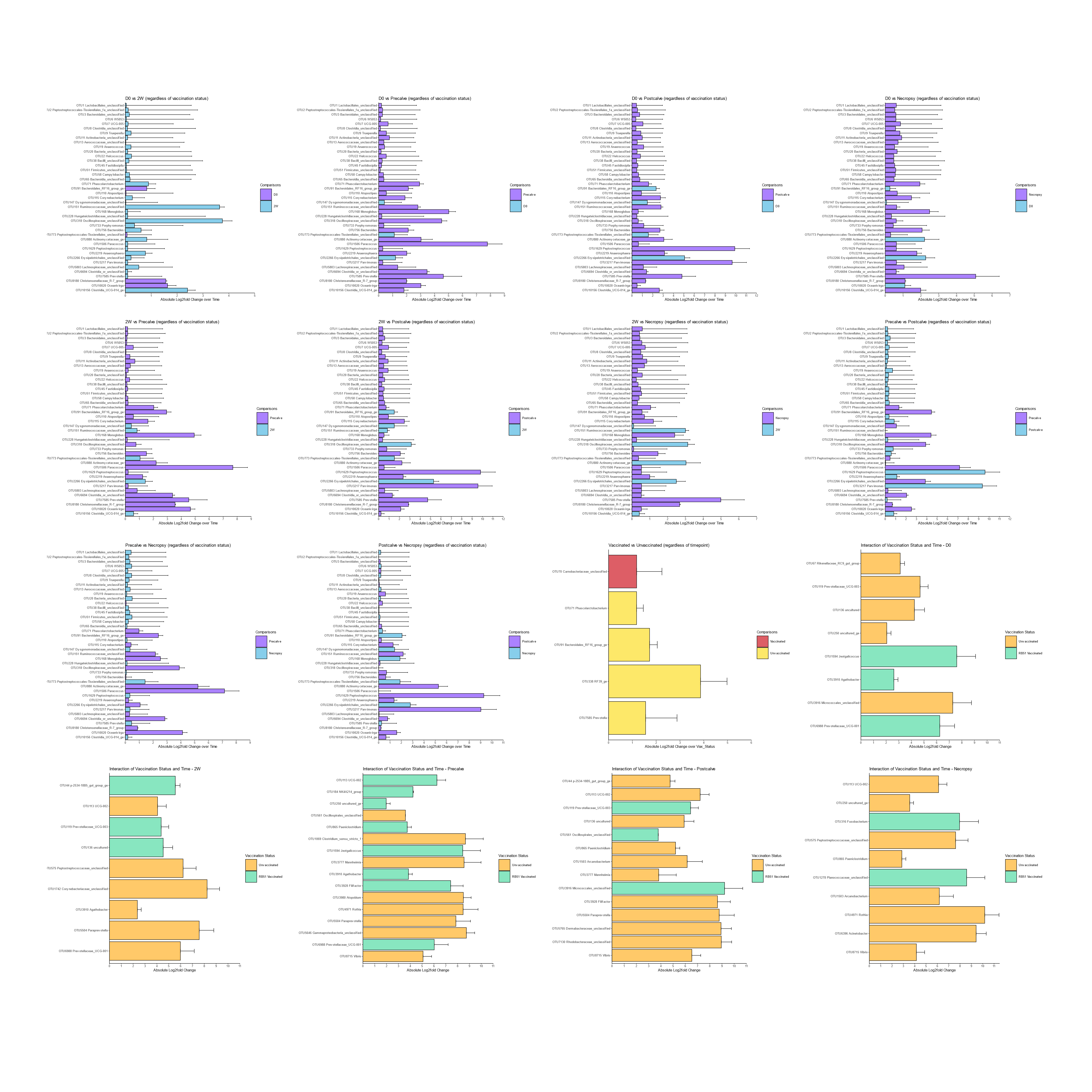

Supplement: SUPPLEMENTARY FIGURE 7 — Vaginal genera which were differentially abundant due to time, vaccination status, or the interaction of time and vaccination status. Differentially abundant taxa are separated by variable of effect (timepoint, vaccination, or interaction effect), and differences in abundance are displayed in absolute log2fold change. [file Image_7.TIF]
